# Why Full Open Access Matters

**DOI:** 10.1371/journal.pbio.1001210

**Published:** 2011-11-29

**Authors:** Michael W. Carroll

**Affiliations:** American University, Washington, D.C., United States of America

## Abstract

This perspective explains the mechanics of copyright and scholarly publishing and warns authors who support open-access publishing about a new pseudo open-access publishing model in which authors pay but publishers still retain commercial reuse rights.

Scientific authors who pay to publish their articles in an open-access publication should be congratulated for doing so. They also should be aware that they may not be getting full open access from some publications that charge for publication under the “open access” label. Two features define an open-access publication: (1) the published contents are freely accessible through the Internet, and (2) readers are given copyright permission (see Box 1) to republish or reuse the content as they like so long as the author and publisher receive proper attribution [1]. Recently, some publications have begun offering an open-access option that charges for Internet publication without granting readers full reuse rights, such as Springer's Open Choice or Nature's Scientific Reports. These publishers have adopted a business model through which authors pay for immediate publication on the Internet but the publisher nonetheless keeps commercial reuse rights for itself. This is not full open access (see Box 2).

**Box 1. A (Very) Brief Primer on Copyright**

For those with an appetite for more details on copyright's mechanics, here goes. Copyright is a set of exclusive rights given to authors to control most reuses of their work without their permission, subject to certain limitations and exceptions to these rights. The theory that justifies copyright is that authors will use these rights either to self-publish or to entice a publisher to remunerate the author and to invest in publishing and distributing the work without fear of unauthorized republication by others. Authors automatically receive copyright at the moment they fix their work in some digital or analog media. (In the United States, old rules required a copyright registration to obtain a copyright, or, later, simply that the work be published with a copyright notice, ©, or copyright would be forfeited. However, since 1978, copyright has been granted automatically at the moment of creation, and since March 1, 1989, copyright has been retained by the copyright owner even if the copyright symbol is not used on publications.)

Under US law, authors can transfer all or part of their copyright if they do so by signing an agreement to this effect. Subscription-based journals usually require authors to transfer all or part of their copyright to the journal as a term of the publication agreement, and usually designate one author as the “corresponding author” who signs on the others' behalf. This is because the subscription model requires publishers to restrict access to paying customers and to use the threat of a copyright infringement lawsuit as a means of deterring competing publications from republishing or reusing the journal's content without a license.

Alternatively, the grant of copyright permission, (also known as a non-exclusive license) can be done verbally or by conduct indicating that permission has been granted. In the case where authors never sign a publication agreement, the publisher has a non-exclusive license and the authors retain copyright.

The open-access model uses this permission model to grant readers broad reuse rights to encourage the widespread republication and reuse of articles. Open-access publishers do not need to police the behavior of readers or rival publishers except to the extent that journal content is reused without giving the author or the journal proper credit. The standard means for granting readers permission is through a Creative Commons Attribution license [3]. (Disclosure: I sit on the Board of Creative Commons.)

With respect to scientific articles, the “author(s)” who get the copyright are sometimes different than the persons listed as authors on a scientific article. Scholarly norms about who receives authorship credit vary by discipline and usually are based on some measure of contribution to a collaborative research undertaking. The extreme case is in high energy physics, in which one article boasts 2,926 authors [9]! In the life sciences this phenomenon usually is related to large-scale clinical trials, such as one article reporting the work of 972 researchers [10].

For copyright purposes, however, authorship is limited to the persons who translate facts and ideas into expression by writing text, creating figures, structuring the data, creating data visualizations, and so on. Within this subset of contributors who count as authors in copyright's eyes, there is one copyright shared equally by the authors responsible for these forms of expression if they had a mutual intent to create an integrated work. Otherwise, if, for example, a figure were created for independent purposes and was then later included in an article, there would be two copyrights owned independently by the respective creators—one in the figure and another in the text.

Traditional copyright is premised on the idea that the authors' and publishers' incentives are aligned because both seek to profit from the publication and distribution of the authors' work. However, this one-size-fits-all approach does not fit scholarly communication—at least in journal form—in which author royalties are the extremely rare exception. Authors write for impact. As scientific publishing has migrated to digital networks, full open access better achieves scientific authors' goals than does the traditional publishing model designed for the production and distribution of printed artifacts.

**Box 2. Requirements for Full Open Access**

*Full open access content is*

* Easily accessible online

AND

* Available to anyone free of charge

AND

* Available for re-use without restriction except that attribution be given to the source.

*No one of these alone qualifies content for an open access label*.

Getting open access right matters because the new publishing model is designed to increase the pace and impact of scientific communication through the power of the Internet. Immediate, free publication increases the audience for scientific research and overcomes the increasingly high price barrier to access imposed by the traditional, subscription-based publishing model [2]. N.B., this audience is comprised of both human readers and their computers, which function more effectively when browsing text on the open web. Liberal reuse rights permit users to republish, quote liberally, and to overcome language barriers through translation [3]. To accomplish these important objectives, the open-access model makes two structural changes to the traditional, subscription-based model. The first is to shift the financing for publication from readers, through subscription fees, to authors (often through their funders), through article processing fees. The second is to shift from a model that uses copyright to control reuse of content to one that uses copyright to encourage republication, preservation, and translation.

## Why Support the Open-Access Financial Model?

Pricing of traditional, subscription-financed scientific journals is highly inefficient. The growth in digital technologies and in digital networks should be driving down the price of access to the scholarly journal literature, but instead prices have increased at a rate greatly in excess of inflation (e.g., [4],[5]). Moreover, studies from journal publishing in some disciplines show that commercial journal publishers successfully charge significantly more than non-commercial journal publishers, such as scholarly societies, even when the commercial offerings make less valuable contributions to the progress of science and knowledge as measured by citations (e.g., [6]).

The economic roots of the pricing problem are not difficult to discern. Journal publishers provide a platform between authors of journal articles and their readers. In these situations, the go-between can choose a mix of prices to each side of the relationship, usually charging more to the party that is more dependent on the go-between. The traditional subscription model charges readers through subscriptions only, but a number of publishers have added page charges or color charges on the author's side as well. Academic and other research-related libraries rather than the readers are the primary purchasers of these journal subscriptions. Journal publishers have extracted generous profits from libraries because their demand is relatively inelastic for two reasons. First, libraries are mission-driven to acquire as broad a swath of the literature as they can afford to serve their patrons effectively. Second, subscriptions for academic journals within a given field are not readily interchangeable, unlike, say, subscriptions to news magazines, because each academic journal publishes unique research. Having their subscribers over a barrel, commercial publishers have steadily consolidated to reduce their costs while increasing profits through uncompetitive pricing [7].

The open-access model fundamentally shifts the balance of power in journal publishing, and thereby greatly enhances the efficiency and efficacy of scientific communication. In its most common form, the model shifts the costs of publication entirely to the author-funder side of the relationship so as to broaden access as far as the Internet reaches and to remove the need for any lingering usage barriers. By shifting the costs of publication entirely to the author-funder side, journals must compete head-to-head on quality and price without diminishing impact through price or usage barriers because authors have greater choice over where to publish than libraries have over whether to subscribe. This increased competition will reduce the overall costs of scholarly communication while broadening access and reuse of the literature.

## Why Support the Open-Access Model for Reader's Rights?

Granting readers full reuse rights unleashes the full range of human creativity to translate, combine, analyze, adapt, and preserve the scientific record, whereas traditional copyright arrangements in scientific publishing increasingly are inhibiting scholarly communication. Traditional copyright law was designed with the subscription-based publishing model in mind. Authors receive copyright when they write their first draft of an article. Authors then transfer this copyright, or grant an exclusive license, to a publisher in exchange for publication. The publisher relies on copyright to police the behavior of readers and competitors who may seek to obtain or redistribute the content without a subscription.

By shifting the financing away from subscriptions, the open-access model realigns copyright to enable broad reuse while assuring authors and publishers that they receive credit for the work they have done. This is done through open licensing by the copyright owner. Initially, the authors of an article automatically own a copyright in the article as soon as it has been drafted. If the authors sign an agreement that transfers the exclusive rights to the publisher, the publisher becomes the copyright owner. The standard means for achieving open access with respect to copyright is for the copyright owner (author or publisher) to use the Creative Commons Attribution license [3], which gives readers and republishers broad reuse rights on the condition that credit for the article is given as directed by whoever is granting the permission. (Disclosure: I sit on the Board of Creative Commons.)

Recently, however, some commercial publishers have waded into the open access waters by charging authors a publication fee to substitute for subscription revenue while limiting reuse. Having been paid for coordinating peer review, editing and laying out the text, and the like, these publishers nonetheless limit readers to making only non-commercial reuses, or even also requiring reusers to use the same license for any adaptations, while reserving to the publisher the rights to make any commercial reuse. (This is done through use of the Creative Commons Attribution Non-Commercial license or the Creative Commons Attribution Non-Commercial Share-Alike license.) This is pseudo open access. Authors who pay for publication in these pseudo open access publications are not getting their money's worth. For example, text or figures subject to these more restrictive licenses cannot be uploaded to Wikipedia, which uses the Creative Commons Attribution Share-Alike license.

Presumably, these publishers retain commercial reuse rights either to derive additional revenues from certain potential reusers or to block competitors, who may exercise these reuse rights to earn revenue through some kind of value-added service or publication. This latter option is possible only if the competitor discovers a market that the original publisher overlooked. Such entrepreneurs should be rewarded rather than controlled.

I suspect that these publishers have commercialized text mining in mind as one of the kinds of reuse they would like to control. This is an illusion. One of the great benefits of open access is that researchers can use any web-based search tool to engage in machine-aided analysis of the published literature. Publishers who lock their content behind a firewall use contracts and technology to limit or prohibit machine-aided research.

Once on the open web, however, even content under a Creative Commons Attribution Non-Commercial license can be freely mined for commercial purposes because the license applies only to uses covered by copyright, and copyright does not regulate text mining—at least in the United States. Copyright attaches only to the author's expression, rather than underlying ideas or facts. The copyright owner has the exclusive rights to reproduce, distribute, adapt, and otherwise communicate the work to the public, subject to certain limitations and exceptions, such as fair use. However, most scientific data are facts that are not covered by copyright except to the extent that an author has exercised minimal creativity in the selection or arrangement of data. This minimal creativity standard might prevent the republication of some tables or figures, but in no case would copyright restrict the reuse of the underlying data if arranged in a different format or in a new figure. Text mining software makes temporary copies of full text. These temporary copies do not count for copyright purposes because of their transitory duration, and the durable outputs of text mining—factual data—are not covered by copyright.

The other use case that may inspire publishers to retain commercial reuse rights is to sell reprints to private sector entities, particularly for life science publishers [8]. It is true that the non-commercial license likely would prohibit redistribution of article copies as advertising for, say, pharmaceutical companies. Full open access could cut into this revenue stream, unless these entities require print copies for which even a full open-access publisher would be free to charge. The commercial publisher may argue that diversifying revenue streams makes good business sense, which it may for them. But, authors, or their funders, should then expect a discount on the publication charge as an offset for these revenues. This approach hinders competition by obscuring journal financing and encouraging accounting gimmicks. It also creates a range of potential roadblocks to future commercial reuses necessary to effective scientific communication.

I offer one example to illustrate the danger, but many others abound. Imagine an evolution in digital formats and a pseudo open-access publisher that has gone bankrupt. The journal's content is on the web but its host site will soon be shut down. A new, for-profit venture sees value in republishing the defunct journal's content in the new format. However, while the journal has died, its copyrights live on (for the life of the author plus another 70 years!). Because the journal demanded the commercial reuse rights even after collecting a hefty publishing fee from the author, the new venture would likely lack the legal right to copy and republish this piece of the scientific record in the new format to the detriment of those authors and the research community at large. We are living through a moment of fundamental opportunity. Let's be clear. Only those publishers willing to fully seize this opportunity deserve to call their publications “open access.”
